# Sancai Lianmei granules ameliorate neuron injury in diabetic ischemic stroke rats by inhibiting oxidative stress and inflammation

**DOI:** 10.3389/fendo.2025.1666597

**Published:** 2025-08-27

**Authors:** Chan Zhu, Dandan Chen, Zhehao Li, Yanmei Wang, Xuke Han, Qiu Chen

**Affiliations:** ^1^ West China Second Hospital of Sichuan University, Traditional Chinese Medicine Department, Chengdu, Sichuan, China; ^2^ West China Second Hospital of Sichuan University, Key Laboratory of Birth Defects and Related Gynecological Diseases, Chengdu, Sichuan, China; ^3^ Shanghai Municipal Hospital of Traditional Chinese Medicine, Shanghai University of Traditional Chinese Medicine, Shanghai, China; ^4^ Ya’an Polytechnic College, Clinical Teaching and Research Department, Ya’an, Sichuan, China; ^5^ Shaanxi University of Chinese Medicine, College of Acupuncture & Tuina, Xianyang, Shaanxi, China; ^6^ Hospital of Chengdu University of Traditional Chinese Medicine, Department of Endocrinology, Chengdu, Sichuan, China

**Keywords:** Sancai Lianmei granules, diabetes, ischemic stroke, traditional Chinese medicine, TLR/NF-κB signaling pathway

## Abstract

**Purpose:**

To assess the impact of Sancai Lianmei (SCLM) granules on diabetic ischemic stroke (IS) model rats, as well as oxygen and glucose deprivation (OGD)-induced PC12 neurons and lipopolysaccharide (LPS)-induced BV2 microglia, and to investigate the associated mechanisms.

**Methods:**

Initially, a diabetic IS model was established in rats through the intraperitoneal administration of niacinamide (NAA) in conjunction with streptozotocin (STZ), supplemented by thread embolization. The model rats were subsequently observed behaviorally, pathologically, and molecularly. Ultimately, the specific mechanism underlying SCLM was elucidated and validated through *in vitro* experiments.

**Results:**

SCLM improved neurological deficits and reduced infarct size in diabetic ischemic stroke models. Furthermore, SCLM modulated the expression of apoptosis-related proteins by downregulating p53 and Bax while upregulating Bcl-2. Additionally, SCLM inhibited the Toll-like receptor 4/nuclear factor kappa-B (TLR4/NF-κB) signaling pathway. *In vitro*, the number of ROS-positive cells and the apoptosis rate were decreased in PC12 cells subjected to OGD and treated with SCLM containing serum, while the LPS-induced inflammatory response of BV2 cells was also alleviated.

**Conclusion:**

The use of SCLM granules is a therapeutic strategy for alleviating oxidative stress and inflammation in diabetic ischemic stroke patients.

## 1 Introduction

Diabetes mellitus (DM) is a global metabolic disorder with rising incidence and mortality (1). According to the International Diabetes Federation, 537 million individuals were diagnosed with diabetes in 2021. Projections estimate that by 2045, the number of individuals with diabetes will increase by approximately 300 million (2).

Ischemic stroke (IS) represents one of the most severe complications associated with DM and is a leading cause of mortality and long-term disability (3). Previous research has identified three distinct characteristics in diabetic stroke patients: 1) Elevated prevalence: the risk of stroke in individuals with DM is 2–4 times higher than that in nondiabetic individuals (4), with an increased risk observable even in the early stages of DM (5); 2) Exacerbated symptoms: due to a higher incidence of cerebral ischemia-reperfusion injury, patients exhibit more severe limb dysfunction and cognitive impairment, as well as an extended hospitalization period (6); and 3) Worse prognosis: the risk of recurrence rate and mortality is higher (7). Consequently, the development of pharmacological treatments for diabetic stroke is crucial to extending survival time and enhancing the quality of life of patients with DM.

Hyperglycemia promotes oxidative stress via multiple pathways. Specifically, hyperglycemia facilitates the reformation of advanced glycation end products, activates protein kinase C, and increases the activity of the hexosamine and sorbitol pathways, ultimately contributing to the development of insulin resistance, impaired insulin secretion, and endothelial dysfunction by inducing excessive reactive oxygen species (ROS) production and oxidative stress (8). Furthermore, a severe imbalance between the excessive production of ROS and the body’s inherent antioxidant capacity exacerbates subsequent oxidative damage in the penumbra of diabetic stroke patients (9).

The stroke process also initiates an inflammatory response that can persist for several months (10, 11). Extensive research has elucidated the complex mechanisms of post stroke neuroinflammation, which contributes to secondary ischemic neuronal damage (12–14). Pharmacological suppression of inflammation has been shown to reduce infarct volume and enhance clinical outcomes in animal models of stroke (15–17). Microglia, as the resident immune cells of the central nervous system, play a crucial role in maintaining tissue homeostasis and contribute to brain development under normal conditions (18). Hyperglycemia induces the activation of overactive microglials, thereby initiating a feed-forward inflammatory loop (19). Notably, the NF-κB signaling pathway is a prototypical proinflammatory signaling mechanism (20). The inhibition of NF-κB can induce apoptosis in aberrantly activated microglial and circulating immune cells, thereby attenuating prolonged abnormal inflammatory responses. This process enhances the inflammatory infiltration microenvironment of the penumbra, supports neuronal survival, and promotes neural regeneration (21, 22).

With its long-standing history and widespread application in the treatment of metabolic diseases, traditional Chinese medicine (TCM) represents a valuable component of Chinese cultural heritage. Sancai Lianmei (SCLM) granules are formulated on the basis of the classical prescriptions Sancai decoction and Lianmei decoction, with modifications as described in the “Detailed Analysis of Epidemic Warm Diseases.” The formulation comprises Renshen (*Panax ginseng C. A. Meyer*), Tian-dong (*Asparagus cochinchinensis (Lour.) Merr.*), Dihuang (*Rehmannia glutinosa (Gaert.)* Libosch*.)*, Wumei (*Prunus mume (Sieb.)* Sieb.), Rougui (Cinnamomum cassia Presl), and Huanglian (*Coptis chinensis* Franch.) (23). Our prior experimental studies revealed that SCLM granules exhibit antioxidant properties that mitigate oxidative stress-induced damage and spermatogenic apoptosis in the testes of mice with type 2 diabetes (24). Moreover, extensive research has demonstrated that the active ingredients of SCLM possess both anti-inflammatory and antioxidative properties (25–27). Consequently, we hypothesized that SCLM might alleviate oxidative stress-induced neuronal dysfunction and subsequent inflammation by regulating the TLR4/NF-kB signaling pathway.

In this study, we first investigated the underlying molecular mechanisms associated with the antioxidative and anti-inflammatory effects of SCLM in Sprague–Dawley (SD) rats subjected to high-glucose conditions combined with middle cerebral artery occlusion followed by reperfusion. Next, we established an oxygen and glucose deprivation (OGD) PC12 cell model to verify the specific antioxidative mechanism by which SCLM improves the oxidation resistance of OGD PC12 cells at the cellular and molecular levels. Finally, we established Lipopolysaccharide (LPS) -induced BV2 microglia and investigated the associated anti-inflammatory molecular mechanisms.

## 2 Materials and methods

### 2.1 Reagents

SCLM granules are derived from the classical Sancai decoction and Lianmei decoction with addition and subtraction in the “Detailed Analysis of Epidemic Warm Diseases,” which consists of Renshen (*Panax ginseng C. A. Meyer*), Tian-dong (*Asparagus cochinchinensis* (Lour.) *Merr.*), Dihuang (*Rehmannia glutinosa* (Gaert.) *Libosch.)*, Wumei (*Pru-nus mume* (Sieb.) *Sieb.*), Rougui (*Cinnamomum cassia Presl*), and Huanglian (*Coptis chinensis Franch.*). SCLM was provided by the Hospital of Chengdu University of Traditional Chinese Medicine. (each gram of SCLM granules contains 1.475 g of raw drug).

### 2.2 Animal experiments

Healthy male Sprague–Dawley rats (n = 60, 6–8 weeks old, weighing 180–220 g) were provided by Beijing HuaFukang Biosciences Co. (INC) (SYXK, 2019–0008, Beijing, China). All animals were maintained under specific pathogen-free conditions in a temperature-controlled room at a constant temperature of 24 ± 1°C with a 12-h light/dark cycle and had free access to a standard rodent diet and water. Following a series of experiments, the rats were anesthetized using an isoflurane inhalation anesthesia machine (3%-4% isoflurane in oxygen) and maintained using continuous mask inhalation (maintenance concentration: 2%). Then, the abdominal aorta was punctured, the circulating blood was drained, and the specimen was collected after perfusion with 4°C saline. All experiments were approved by the Ethical Committee for the Experimental Use of Animals at the Hospital of Chengdu University of Traditional Chinese Medicine (2021SDL-004).

#### 2.2.1 DM model establishment and pretreatment

“After 1-week acclimation, rats were randomized into control (n=10) and DM groups (n=50). The DM group received 100 mg/kg NAA (i.p.), followed by 65 mg/kg STZ in citrate buffer (pH 4.5, i.p.) The normal group was injected with an equal volume of citric acid buffer. Blood samples were obtained from the tail vein 24 hours after STZ injection for blood glucose measurement to confirm hyperglycemia (28, 29). Rats with blood glucose concentrations greater than 17.6 mmol/L (n = 50) were randomly divided into five groups (n = 10/group): the vehicle group, SCLM low-dose (SCLML, 3.4 g/kg/d), medium-dose (SCLMM, 6.8 g/kg/d), high-dose (SCLMH, 13.6 g/kg/d) and metformin groups (Met, 250 mg/kg/d). Body weight, water intake and fasting blood glucose levels were measured every 2 weeks for 8 weeks. Rats in the control group and vehicle group were given equal volumes of normal saline. All drugs were administered intragastrically from day 1 to day 7. SCLM-containing blood was collected from the different groups after dosing at 1 h after the last dose. The blood was subsequently centrifuged at 4°C (12,000 rpm-10 min) to obtain the upper layer of serum. The serum was separated and stored at -80 °C for use in cell experiments. The bacteria were removed via a 0.22 μm filter for subsequent experiments.

#### 2.2.2 Rat stroke model establishment and evaluation

Briefly, middle cerebral artery occlusion/reperfusion (MCAO/R) model rats were generated by inserting a filament (MSRC35B200PK50, RWD Co., Ltd., China) with a silicone tip into the middle cerebral artery of SD rats for 90 minutes, after which the filament was removed to allow reperfusion. In the control group, only the skin and muscles were scratched without embolization or treatment.

##### 2.2.2.1 Neurological deficit score

Neurological evaluations of MCAO rats were conducted via a modified neurological severity score 24 hours post reperfusion. The assessment protocol comprised six distinct tests evaluating spontaneous activity, symmetry in limb movement, four limbs, forepaw extension, climbing ability, body proprioception, and response to vibrissae stimulation. Each rat underwent this comprehensive neurological assessment, and the cumulative score was derived from the sum of the individual test scores. Importantly, the investigators conducting the assessments were blinded to the group assignments to ensure unbiased evaluation.

##### 2.2.2.2 Infarct size measurement

The brain tissues (n = 3 per group) were removed and placed in the freezer for 20 min, after which they were cut into five 2-mm slices (from rostral to caudal). Then, the brain slices were added to 2% 2,3,5-triphenyltetrazolium chloride (TTC) solution (Solarbio, China) and incubated at 37°C for 20 minutes. After TTC staining, the brain slices were fixed in 4% paraformaldehyde solution for imaging and quantification analysis. The images were quantitatively analyzed via ImageJ software (National Institutes of Health, USA).

### 2.3 Biochemical analysis

Blood was collected via abdominal aorta, centrifuged (300 ×g, 10 min), and serum stored at −80°C. The levels of alanine aminotransferase (ALT), aspartate aminotransferase (AST), creatinine (Cr) and albumin (ALB) in the serum were detected via an automatic biochemical analyzer (URIT-8021A, Guilin Youlite Medical Electronic Co., Ltd.).

### 2.4 Histopathological examination

The brain tissues were fixed with 10% neutral formalin for 48 hours immediately after harvest and then rinsed with water for 30 min. Then, the brain tissues were dehydrated, cleared, penetrated in paraffin in an automatic dehydrator (EXCELSIOR AS, Thermos) and then embedded in paraffin (HISTOSTAR, Thermos). The continuous sliced histological sections were prepared using HM 340E (Thermos) with a thickness of 4 μm. A standard procedure for staining soft tissue with hematoxylin–eosin (H&E) and Nissl staining was performed to demonstrate the general histological structure of the tissues.

### 2.5 Immunohistochemical analysis

The sections were blocked with 10% bovine serum at room temperature for 1 hour and incubated overnight at 4°C with the following primary antibodies: rabbit monoclonal to Iba-1 (ab178846, 1:2000 dilution, Abcam, UK) and rabbit polyclonal to NeuN (ab104224, 1:250 dilution, Abcam, UK). Afterwards, the sections were incubated with horseradish peroxidase (HRP)-labeled secondary antiserum (DAKO EnVisonTM kit) at 37°C for 30 min. Immunoreactivity was identified with 0.05% diaminobenzidine, and the sections were visualized with an inverted fluorescence microscope (#IX83, Olympus, Tokyo, Japan). Three randomly selected microscopic fields were analyzed in the ischemic penumbra at the level of the bregma. The numbers of Iba-1-positive and NeuN-positive cells were calculated via ImageJ software.

### 2.6 Cell culture

PC12 and BV2 lines were purchased from Procell Life Science & Technology Co., Ltd. PC12 cells were incubated in RPMI 1640 medium supplemented with fetal bovine serum (FBS) (10%), and BV2 cells were incubated in DMEM supplemented with FBS (10%) at 37°C in a 5% CO_2_ incubator and passaged according to the manufacturer’s instructions. The cells were seeded in plates at a density of ~10^5^ cells mL^-1^ and cultured overnight prior to the experiments.

#### 2.6.1 Cell Counting Kit-8 assay

After incubation for 24 hours, the number of viable cells was determined with an Enhanced Cell Counting Kit-8 (CCK-8) (#C0041, Beyotime, Jiangsu, China) according to the manufacturer’s instructions. Absorbance at 450nm was measured and normalized to controls.

#### 2.6.2 *In vitro* modeling of DM

The PC12 cell DM model was induced with different concentrations of high glucose (HG). The HG was diluted with complete RPMI 1640 medium to 25, 50, and 75 mM. The appropriate concentration of HG was systematically evaluated via a CCK-8 kit.

#### 2.6.3 *In vitro* modeling of ischemic stroke

OGD was used to simulate ischemic injury *in vitro*. The cells were cultured in glucose-free medium in an incubator at 37°C for 0, 2, 3, 4, 6, 8, 12 hours in a 95% N_2_/5% CO_2_ gas mixture to mimic hypoxic conditions. Then, the cells were transferred to normal culture medium for an additional 2 hours at 37°C under 5% CO_2_ for reoxygenation. The appropriate duration of oxygen deprivation was systematically evaluated via a CCK-8 kit.

#### 2.6.4 *In vitro* modeling of inflammation

LPS-stimulated BV2 cells constitute a widely recognized *in vitro* inflammatory model (30). In our study, when BV2 cells reached the appropriate density, 1 μg/mL LPS was added to the medium for 24 h.

### 2.7 Grouping of the therapeutic effects of SCLM *in vitro*

To evaluate the therapeutic effect of SCLM on HG+OGD-induced neuronal injury, PC12 cells were cult*u*red in HG+OGD medium as a model group and incubated in complete RPMI 1640 medium supplemented with 10% normal serum as a control group. The HG+OGD group was treated with low, moderate, or high concentrations of SCLM drug-containing serum for 24 hours. FBS group cells were incubated in complete RPMI 1640 medium with 10% FBS as a negative control.

### 2.8 Apoptosis analysis

Flow cytometric analysis was used to determine the extent of PC12 apoptosis induced by HG+OGD and the recovery ability of SCLM. Briefly, PC12 cells (1.5×10^5^/mL) were plated in 12-well plates, treated with SCLM for 4 h after adherence. Next, the cells were incubated with the ROS solution for another 20 min. After being removed from the medium and washed with cold phosphate-buffered saline (PBS), the cells were subsequently stained with annexin V and PI according to the experimental methods of the assay kit (FXP018, 4A Biotech) specification. Apoptosis was analyzed via a Beckman CytoFLEX S flow cytometer. According to the fluorescence intensity, the cell populations were assigned to four quadrants: live (Q4), early apoptotic (Q3), late apoptotic (Q2), and necrotic (Q1) cells.

### 2.9 Western blot analysis

Immunoblot detection was carried out using the standard protocol. The antibodies used for immunoblotting were as follows: TLR4/Toll-like receptor 4 rabbit polyclonal antibody (cat.no. A8187, Beyotime), phospho-IκB alpha (Ser32) rabbit monoclonal antibody (cat.no. AF187, Beyotime), phospho-NF-κB p65 (Ser276), rabbit polyclonal antibody (cat.no. AF587, Beyotime), Bax rabbit monoclonal antibody (cat.no. AF1270, Beyotime), Bcl-2 rabbit polyclonal antibody (cat.no. AF6285, Beyotime), IL1B rabbit polyclonal antibody (cat.no. AF720, Beyotime), and anti-p53 (#C0041, Beyotime). β-Actin polyclonal antibody (cat. no. 20536-1-AP; Proteintech, USA), HRP-labeled goat anti-rabbit IgG (H+L) (cat. no. A0208, Beyotime) was used as a reference.

### 2.10 Evaluation of intracellular ROS

Briefly, PC12 cells (2.5×10^5^/mL) were seeded in 12-well plates, treated, and stained with DCFH-DA (20 min). Meanwhile, in the blank control group, no ROS solution was added, and the other experiments were the same as those in the experimental group. After washing with PBS, the ROS-positive cells were observed and recorded under an inverted fluorescence microscope (#IX83, Olympus, Tokyo, Japan), and the positive cell counts were calculated via ImageJ software.

### 2.11 TUNEL assay

TUNEL was conducted with a DMK500 *in situ* apoptosis kit (TAKARA BIO Inc., Shiga, Japan) according to the manufacturer’s instructions. In brief, 3 mm-thick coronal brain sections were immersed in a TdT-labeling reaction mixture for 90 min, followed by DAB staining for nearly 5 min and anti-FITC HRP counterstaining for 30 min at 37°C. TUNEL-positive cells in the ischemic penumbra of a coronal section were identified with an inverted fluorescence microscope (#IX83, Olympus, Tokyo, Japan). The number of TUNEL-positive cells in the ischemic penumbra from three randomly selected fields was determined via ImageJ software. The apoptosis index is expressed as the percentage of TUNEL-positive cells among the total number of cells per field of view.

### 2.12 Statistical analysis

GraphPad Prism version 8 (GraphPad Software Inc., CA, USA) was used for statistical analysis. To compare two groups, we used a two-tailed Student’s t test. To compare multiple groups, we used one-way analysis of variance (ANOVA). n.s., not significant, *P < 0.05; **P < 0.01; ***P < 0.001; ****P < 0.0001. The data are presented as the means ± standard deviations (SDs) if n ≥ 3.

## 3 Results

### 3.1 Therapeutic effects of SCLM on rats

#### 3.1.1 Effects on glucose, body weight and water intake in DM rats

**Figure 1:**
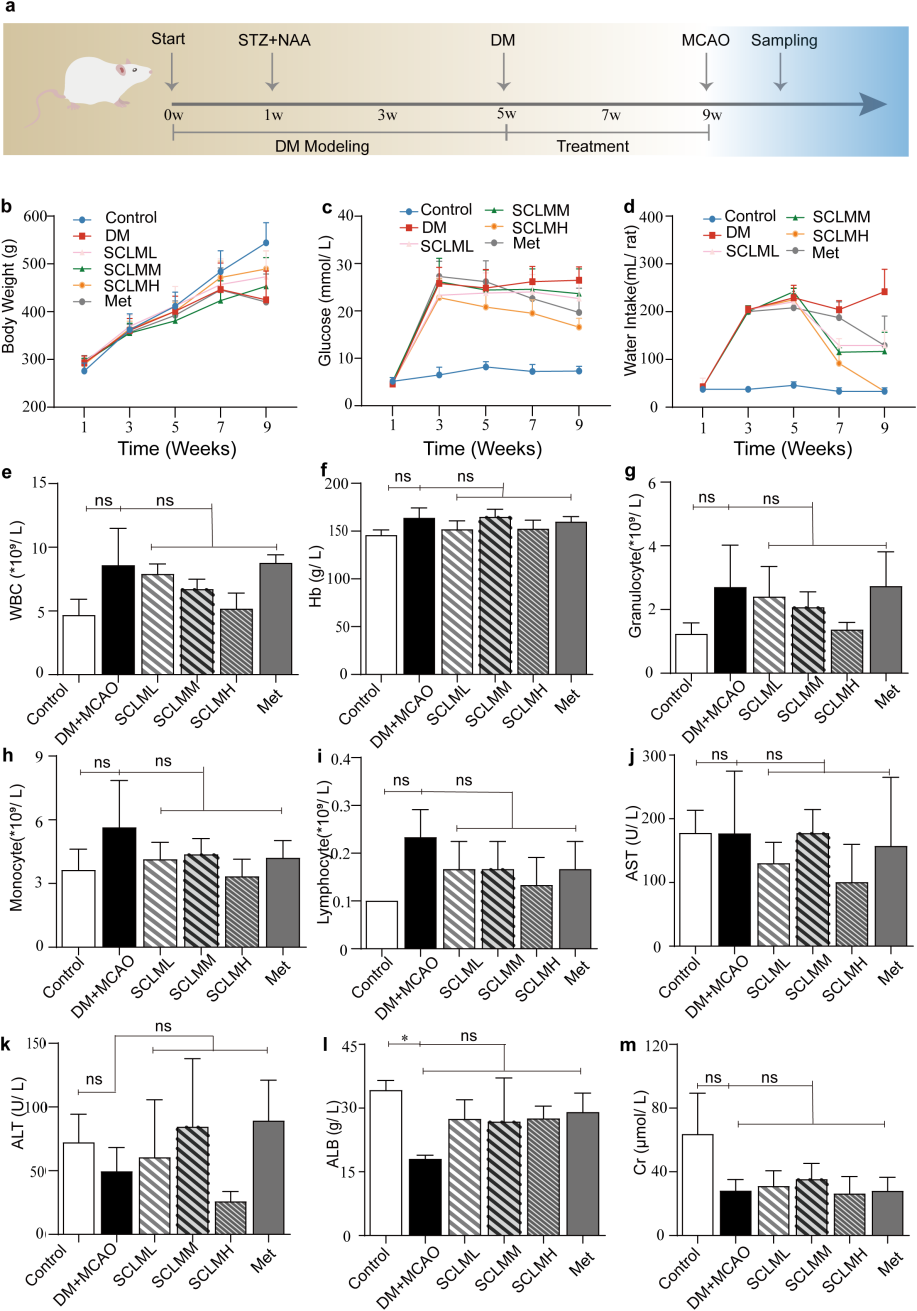
Therapeutic effects of SCLM on rats. **(a)** Experimental design for the establishment, therapy and examination of the DM + MCAO/R rat model. **(b-d)** Body weight and glucose and water intake in different groups over 8 weeks. **(e-i)** Major markers of routine blood examination in different groups 24 hours after MCAO. **(j-m)** The concentrations of AST, ALT, ALB, and Cr in the serum of rats in different groups 24 hours after MCAO.

To investigate the effects of SCLM on glucose and body weight in diabetic rats, we used NAA combined with STZ to establish a T2DM model in SD rats (**Figure 1a**). Body weight and fasting blood glucose (FBG) levels were recorded every two weeks during the experiment. Compared with those of the control group, the body weights of the DM rats were significantly lower (**Figure 1b**, **Supplementary Table S1**), and blood glucose and water intake increased significantly (**Figures 1c, d**, **Supplementary Tables S2, 3**). SCLM treatment alleviated weight loss in DM rats to varying degrees and reduced the FBG of DM rats compared with those in the model group, but these differences were not statistically significant (**Figures 1b, c**, **Supplementary Tables S1, S3**). Interestingly, water intake decreased significantly in the SCLM treatment group (**Figure 1d**, **Supplementary Table S2**).

### 3.2 Effects on systemic circulation and cerebral tissue in DM/MCAO rats

We detected nine serum indicators in rats (**Figures 1e–m**) after MCAO. Compared with the control group, the DM/MCAO group presented significantly lower levels of ALB (**Figure 1i**).

**Figure 2:**
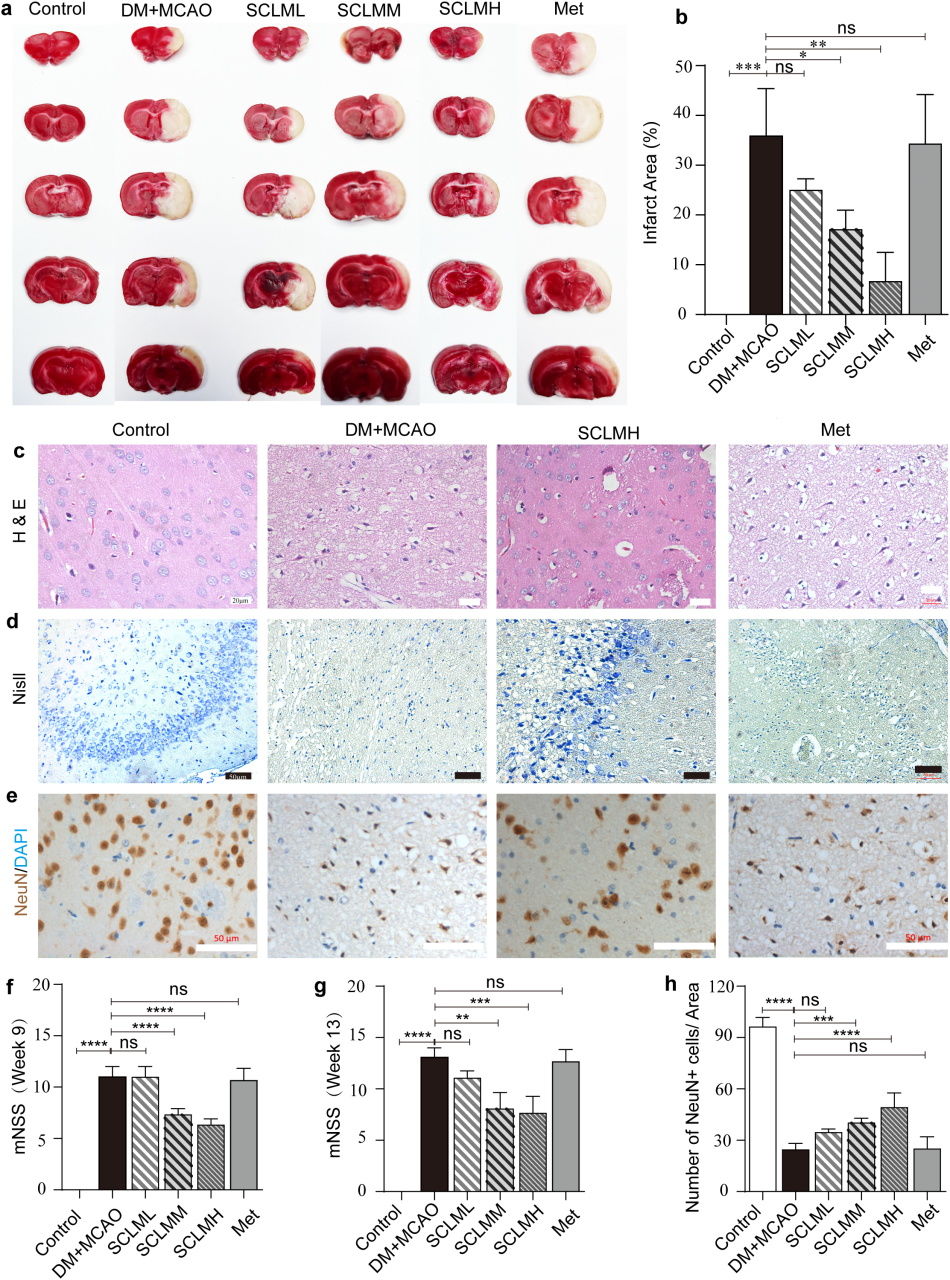
*In vivo* antagonism of cerebral ischemic-reperfusion injury by SCLM. **(a)** Representative images of TTC-stained brain slices of the indicated groups; the red represents normal area and the white represents infarct area. **(b)** Infarct ratio of different groups analyzed by Image J (n = 3 per group). **(c)** Representative brain tissue photomicrographs of H&E and **(d)** Nissl staining from different treatment groups. **(e)** NeuN(brown) and DAPI (blue) co-staining of MCAO/R rats brain sections. **(f)** Neurological scores of MCAO/R rats in week 9 (n = 12 per group) and week 13(n = 3 per group). **(h)** Corresponding semiquantitative analysis of **(e)**(n = 5 per group). P values are assessed by two-way ANOVA and marked as asteroids. ****P < 0.0001 and ns represents no significant difference.

The neurological scores and infarct areas of the stroke rats were analyzed after pretreatment with SCLM for 4 weeks (**Figure 1a**). As shown in **Figure 2a**, the stroke rats that were administered saline (DM+MCAO group) exhibited a significant area of infarction, which was reflected by the inability to stain with TTC solution, whereas pretreatment with SCLM in stroke rats significantly decreased the brain infarct area, and this effect was concentration dependent (**Figure 2b**). Furthermore, the neurological scores of stroke rats improved after pretreatment with SCLM (**Figure 2f**), and the therapeutic effect was maintained long-term (**Figure 2g**). We also elucidated the therapeutic effects of SCLM as a neuroprotective agent via pathological analysis of brain tissue. First, H&E staining of brain sections from stroke rats revealed that the saline-injected group exhibited a significant decrease in neurons with irregular morphology and disordered arrangement, indicating severe neuronal damage. In contrast, pretreatment with SCLM notably decreased the necrotic area (**Figure 2c**). We subsequently examined neuronal damage in the infarcted area of stroke rats via Nissl staining. As shown in **Figure 2d**, a noticeable reduction in Nissl bodies was observed in the infarcted area of stroke rats that received saline injection compared with the sham-operated control group. However, after MCAO rats were pretreated with SCLM, the number of Nissl bodies in ischemic brain tissue increased significantly. Additionally, compared with those in the control group, the number of NeuN^+^ cells in the model group was significantly lower, whereas the number of NeuN^+^ cells in SCLM-pretreated stroke rats was greater than that in saline-treated stroke rats in a dose-dependent manner (**Figures 2e,h** and **Supplementary Figure S1**).

### 3.3 SCLM alleviates neuron apoptosis by inhibiting oxidative stress

**Figure 3:**
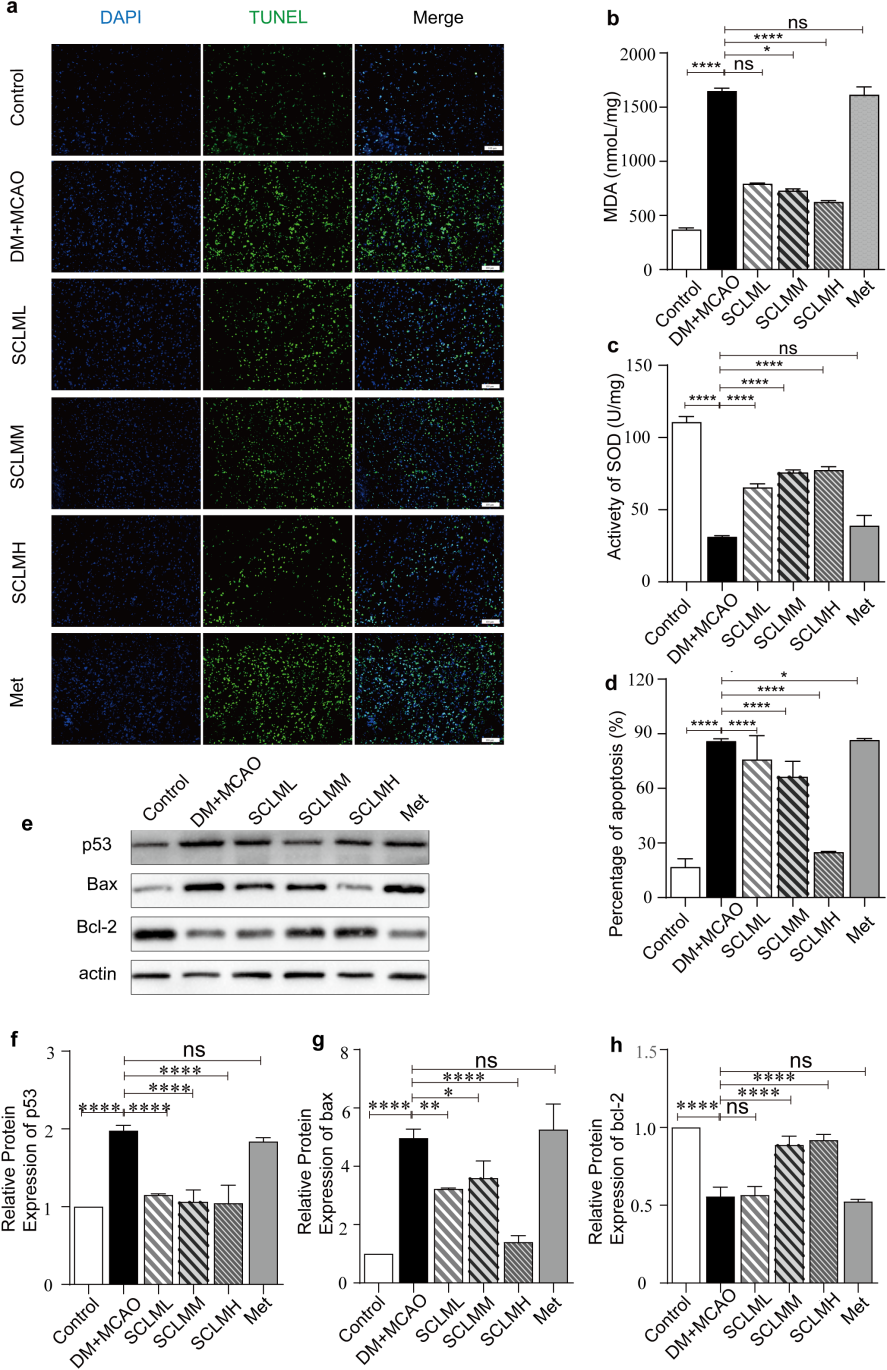
SCLM inhibits oxidative stress response and apoptosis *in vivo*. **(a)** TUNEL (green), and DAPI (blue) co-staining of MCAO/R rats brain sections. **(b)** Expression levels of MDA and **(c)** SOD in the brain tissue of different treatment groups (n = 6 per group). **(c)** Representative brain tissue photomicrographs of H&E and **(d)** Corresponding semiquantitative analysis of **Figure 3a** (n = 5 per group). **(e)** Representative Western blots of p53, Bax and Bcl-2 protein of PC12 cells in different groups. Corresponding quantitative analysis of **(f)** p53, **(g)** bax and **(h)** bcl-2 protein. P values are assessed by two-way ANOVA and marked as asteroids. *P < 0.05, ****P < 0.0001 and ns represents no significant difference.

Oxidative stress plays an important role in the pathogenesis of ischemic stroke. Using both *in vivo* DM/MCAO rat models and *in vitro* experimental systems, we demonstrated that SCLM alleviates neuronal apoptosis by inhibiting oxidative stress. Malondialdehyde (MDA) and superoxide dismutase (SOD) levels are important indicators for evaluating oxidative stress damage. Therefore, MDA and SOD levels of brain tissues were detected in this study to evaluate the antioxidant function of SLCM in DM/MCAO rats. Compared with that in the control group, the MAD content increased significantly (**Figure 3b**), whereas the activity of SOD decreased in the DM/MCAO group (**Figure 3c**). Compared with the DM/MCAO group, the SCLM group presented a significantly increased MDA clearance rate and SOD activity. These results indicate that SCLM inhibits lipid peroxidation in the brain tissues of DM/MCAO rats by exerting SOD enzyme-like activity. In addition, immunostaining via terminal deoxynucleotidyl TUNEL in brain sections revealed that the number of TUNEL^+^ cells in SCLM-treated rats was lower than that in saline-treated stroke mice, indicating that SCLM treatment likely contributed to protection against neuronal apoptosis (**Figures 3a, d**).

Oxidative damage, which is triggered by the overproduction of ROS, is critical in contributing to damage in ischemic brain tissues. Therefore, we investigated the effects and mechanisms of SCLM against ROS. The upregulation of proapoptotic p53 and bax and the downregulation of antiapoptotic Bcl-2 are associated with ischemia-induced cell death/apoptosis, whereas the upregulation of Bcl-2 can suppress the generation of ROS. We conducted Western blot studies with brain tissues to assess whether the regulation of the p53, Bax and Bcl-2 proteins is involved in the neuroprotective effect of SCLM *in vivo*. As shown in **Figure 3e**, in ischemic penumbra brain tissues, compared with the control group, the DM+MCAO group presented increased expression of proapoptotic p53 and Bax and decreased expression of antiapoptotic Bcl-2. Conversely, SCLM pretreatment explicitly downregulated the expression of proapoptotic p53 and bax proteins and upregulated the expression of antiapoptotic Bcl-2 proteins (**Figures 3f–h**).

PC12 cells have been used as a common *in vitro* neuronal model to explore the neuroprotective effects of novel pharmaceuticals (31). OGD has been extensively used as an available method to induce a hypoxic microenvironment and subsequently overproduce ROS in different cell lines and has been deemed a common *in vitro* model for exploring the mechanism of antioxidants against ischemia-associated disorders (32).

First, a CCK-8 assay was used to investigate the toxicity of SCLM to PC12 cells. (**Supplementary Figure S2**). Different concentrations of glucose were subsequently used to establish HG models, and mannitol (at the same concentration as HG) was used to eliminate the effect of osmotic pressure on the cells (**Supplementary Figure S3**). Finally, the duration of hypoxia was determined. We found that additional glucose did not significantly affect cell viability, whereas 3 hours of hypoxia resulted in a significant increase in cell viability (**Supplementary Figure S4**). Therefore, this condition (HG: 50 mmol/L, OGD: 3 h) was used in the subsequent experiments.

**Figure 4:**
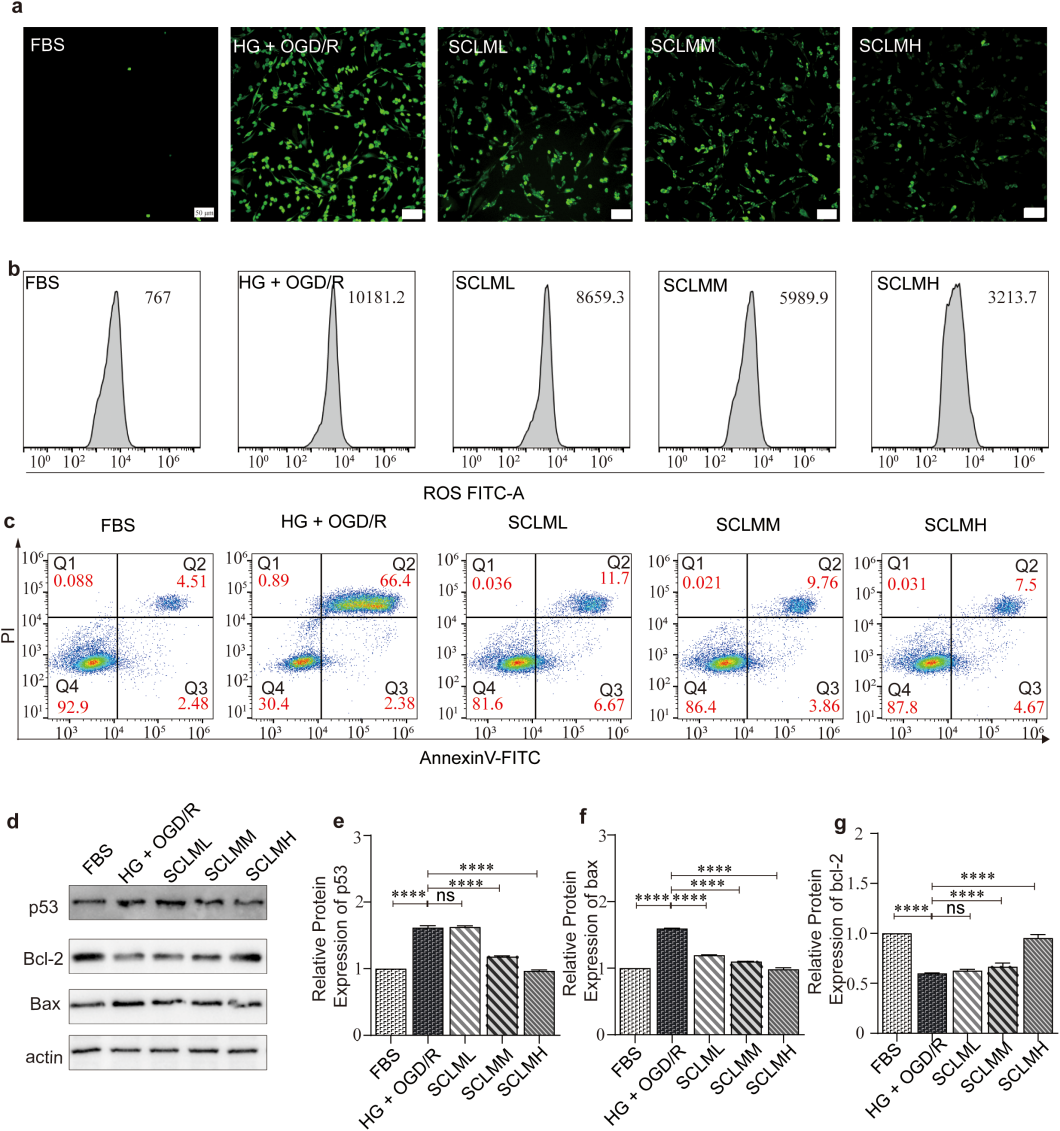
Protection of SCLM to PC12 cells by through inhibiting oxidative damage and apoptosis. **(a)** Fluorescent images of general ROS detected in in hypoxic condition damaged PC12 using 2′,7′-dichlorofluorescin diacetate **(DCFH-DA)** probe. Scale bars, 50 μm. **(b)** Quantification of ROS level by the flow cytometer detected in hypoxic condition damaged PC12 using DCFH-DA probe. **(c)** Annexin V/PI-labeled flow cytometry assay of PC12 in different conditions. **(d)** Representative Western blots of p53, Bax and Bcl-2 protein of PC12 cells in different groups. Corresponding quantitative analysis of **(e)** p53, **(f)** bax and **(g)** bcl-2 protein. Each protein expression level is normalized to the expression of β-actin. Data are presented as mean ± SD. P value are assessed by one-way ANOVA analysis. Asterisks indicate p values *P < 0.05, **P < 0.01,****P < 0.0001, and ns represents no significant difference.

The free radical scavenging ability of SCLMs in PC12 cells was first measured by DCFH-DA. PC12 cells treated with HG+OGD were used as a free radical positive control. As shown in **Figure 4a**, compared with those in the HG+OGD group, the free radical levels in the SCLM groups significantly decreased in a dose-dependent manner. Furthermore, we used flow cytometry to verify the fluorescence intensity of the ROS levels in PC12 cells after treatment with SCLM (**Figure 4b**).

ROS play important roles in the induction of apoptosis. Therefore, we performed an Annexin V and propidium iodide costaining assay to examine the reversal of ROS-induced apoptosis by SCLM. Similarly, SCLM decreased apoptosis in HG+ODG-stimulated PC12 cells in a dose-dependent manner (**Figure 4c**).

We also performed Western blot analyses on PC12 cells to investigate whether the regulation of the p53, Bax and Bcl-2 proteins contributes to the neuroprotective effects of SCLM *in vitro*. As shown in **Figures 4d–g**, PC12 cells subjected to high glucose and hypoxic conditions presented elevated levels of the proapoptotic proteins p53 and Bax, which are long-term proteins that reduce the level of the antiapoptotic protein Bcl-2, compared with those in cells not exposed to HG+OGD. In contrast, pretreatment with SCLM significantly downregulated the expression of the proapoptotic proteins p53 and Bax while increasing the expression of the anti-apoptotic protein Bcl-2.

These findings indicate that SCLM can exert antiapoptotic effects to protect cells from ischemia-induced injury by regulating the expression of anti- and proapoptotic proteins.

### 3.4 SCLM suppresses inflammation and immune response by inhibiting TLR4/NF-κB signaling pathway

**Figure 5:**
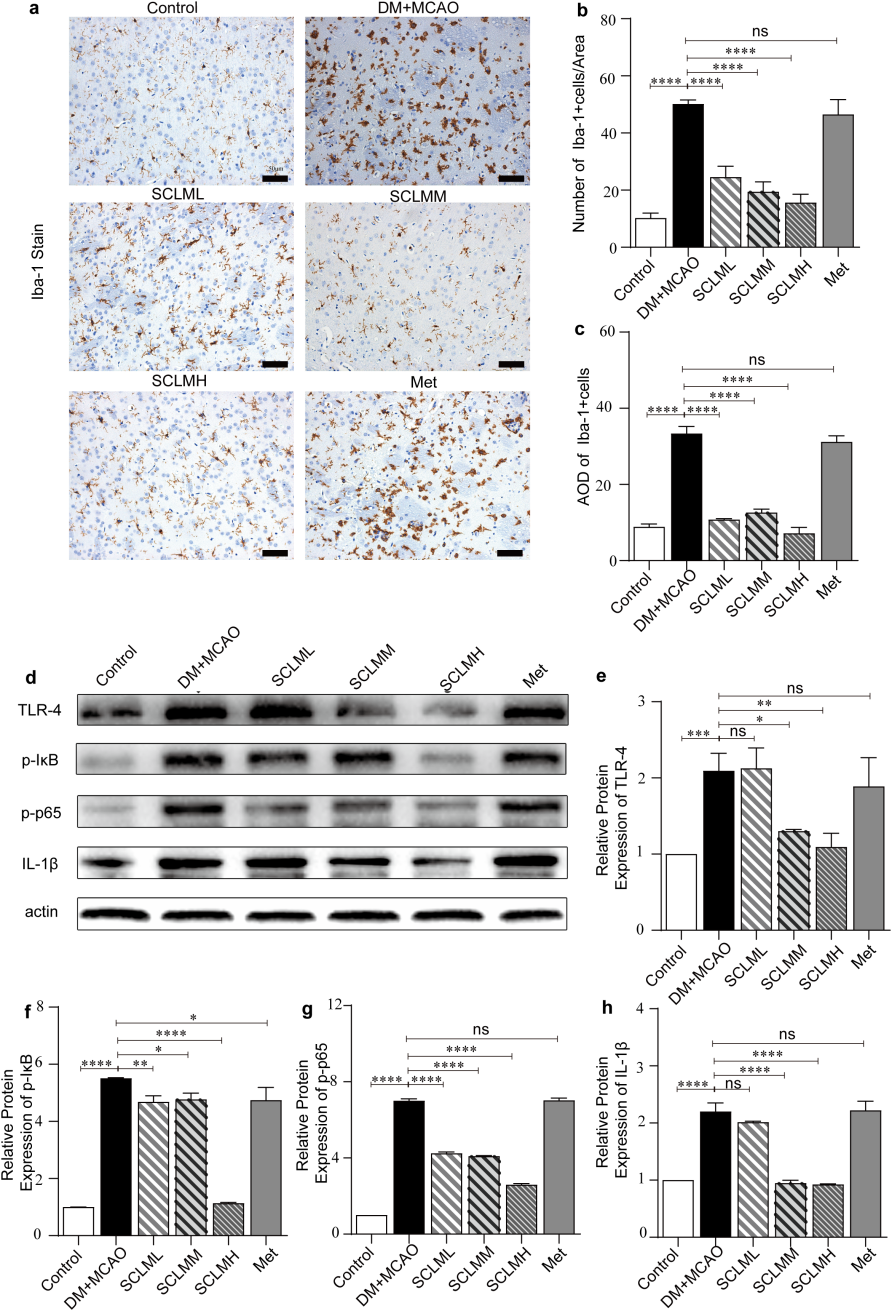
Anti-inflammatory effects of SCLM *in vivo*. **(a)** Iba-1 staining of rats with different treatments and their semiquantity analysis displayed in **(b, c)**. Scale bars, 50 μm. **(d)** Representative Western blots of pro-inflammatory cytokines IL-β, RL-4 and NF-κB in ipsilateral hemispheres from different groups. Corresponding quantitative analysis of **(e)** TRL-4, **(f)** pI-κB, **(g)** p-p65 and **(h)** IL-1β protein. Each protein expression level is normalized to the expression of β-actin. Data are presented as mean ± SD. P value are assessed by one-way ANOVA analysis. Asterisks indicate p values *P < 0.05, **P < 0.01,***P < 0.001****P < 0.0001, and ns represents no significant difference.

The inflammatory response is recognized as another crucial contributor to cell death after ischemic stroke. Immune cells in brain, microglia, can be dramatically activated following ischemia, which leads to secretion of inflammatory mediators and further contributes to the invasion of peripheral inflammatory cells (33). These, in turn, can further exacerbate neuronal injury in the cerebral ischemic penumbra and exaggerate damage to the surrounding regions. As observed in this study, DM+MCAO markedly increased the number of Iba-1-positive cells in the ischemic penumbra (**Figures 5a–c**). However, these alterations were significantly attenuated in SCLM-pretreated DM+MCAO rats.

**Figure 6:**
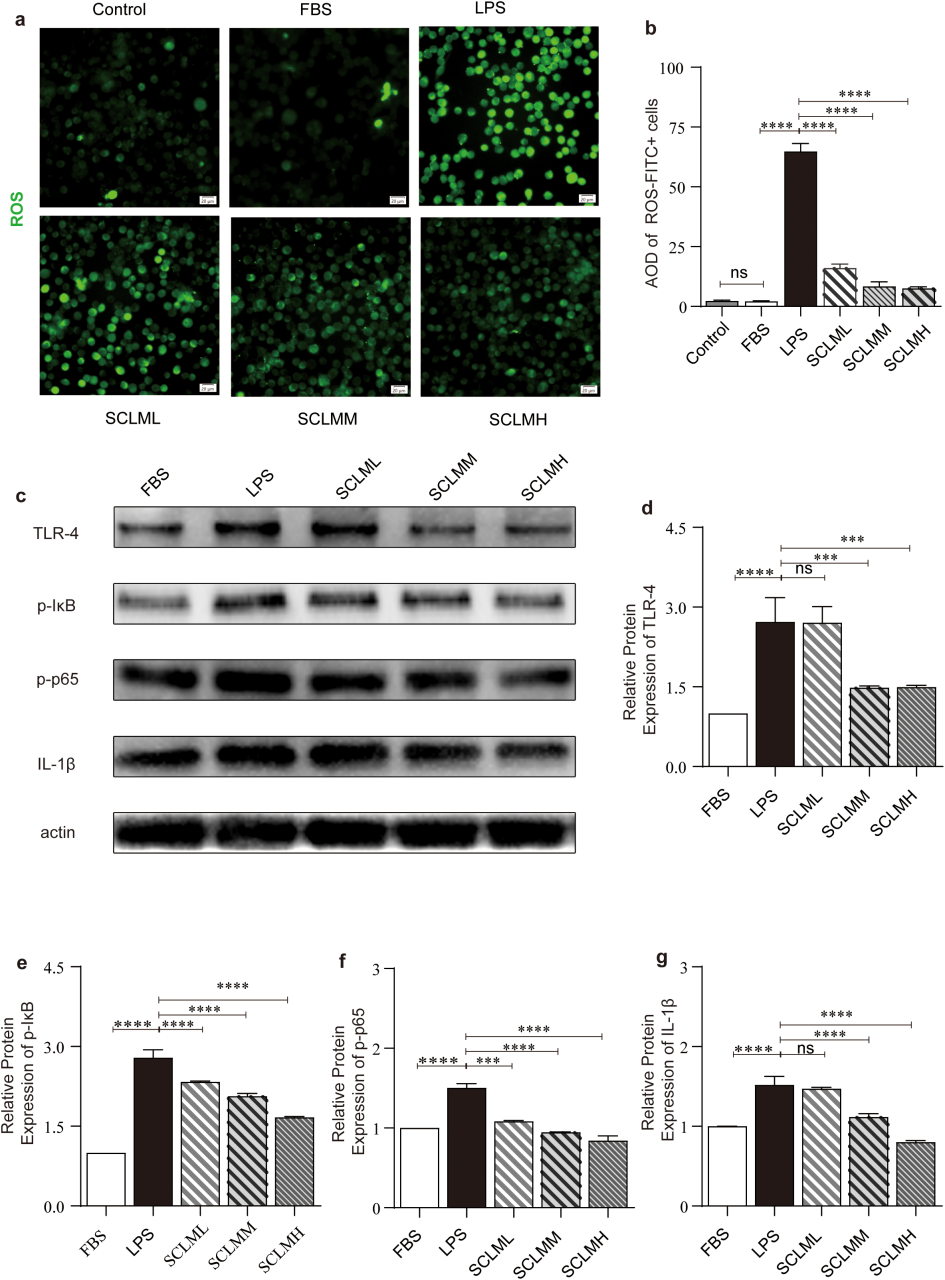
Anti-inflammatory effects of SCLM *in vitro*. **(a)** Fluorescent images of general ROS detected in in LPS-stimulated BV2 using 2′,7′-dichlorofluorescin diacetate **(DCFH-DA)** probe. Scale bars, 20 μm. **(b)** Quantification of ROS level by the flow cytometer detected in hypoxic condition damaged BV2 using DCFH-DA probe. **(c)** Representative Western blots of pro-inflammatory cytokines IL-1β, TRL-4 and NF-κB from different groups. Corresponding quantitative analysis of **(d)** TRL-4, **(e)** pI-κB, **(f)** p-p65 and **(g)** IL-1β protein. Each protein expression level is normalized to the expression of β-actin. Data are presented as mean ± SD. P values are assessed by one-way ANOVA analysis. Asterisks indicate p values ***P < 0.001, ****P < 0.0001, and ns represents no significant difference.

NF-κB proteins are a family of transcription factors that are of central importance in inflammation (21). ROS plays a vital role in NF-κB signaling (34). In addition, activation of microglia has been reported to contribute to the generation of ROS (35). To determine whether SCLM confers cellular protection through its anti-inflammatory properties, we examined the expression levels of proinflammatory mediators and cytokines in brain tissues. As illustrated in **Figures 5d–h**, SCLM significantly inhibited the expression of TLR4, p-IκB, p-p65, and IL-1β in the brain tissues of DM+MCAO rats. For *in vitro* experiments, we utilized LPS-stimulated BV2 cells, a widely recognized *in vitro* inflammatory model derived from C57BL/6 mouse microglia. First, the toxicity of SCLM in BV2 cells was evaluated, and similarly, no obvious cytotoxicity was detected (**Supplementary Figure S5**). As shown in **Figures 6a, b**, compared with those in the LPS group, the free radical levels in the SCLM group significantly decreased in a dose-dependent manner. Then, we detected the expression levels of proinflammatory mediators and cytokines in LPS-stimulated BV2 cells using western-blot. The results showed that SCLM inhibited the protein expression of TLR4, p-IκB, p-p65 and IL-1β in LPS-stimulated BV2 cells (**Figures 6c–g**). Similarly, these findings suggest that SCLM pretreatment may reduce inflammation by inhibiting NF-κB activation, thereby decreasing the release of proinflammatory mediators and cytokines after ischemic injury.

## 4 Discussion

Diabetes, a prevalent metabolic disorder, promotes vascular lesions through metabolic abnormalities and oxidative stress, leading to lipid accumulation, inflammation, extracellular matrix deposition, and endothelial dysfunction, ultimately impairing micro- and macrovascular function (36, 37). The deleterious effects of diabetes on ischemic stroke have been well-documented, identifying it as a significant risk factor for adverse clinical outcomes, including increased mortality (38). In this study, the neuronal function of DM+MCAO mice was impaired, and the infarct area was significantly enlarged. However, these effects were reversed by SCLM treatment. These findings suggest that SCLM may reduce the infarct area, thereby alleviating damage to neuronal function.

Current evidence indicates that apoptosis in the penumbra is closely associated with oxidative stress (39). The diabetes drug semaglutide can reduce infarct size, inflammation, and apoptosis and normalize neurogenesis in a rat model of stroke (40). Berberine, a primary pharmacologically active constituent of *Coptis chinensis*, can inhibit neuronal apoptosis in cerebral ischemia (41). In this study, we observed a significant increase in the level of apoptosis in the penumbra of DM+MCAO rats compared with that in the sham group. The apoptosis index was notably inhibited by SCLM, suggesting that SCLM modulates excessive neuronal apoptosis in the penumbra of SD+MCAO and thereby protects neuronal function. In addition, the p53 gene is recognized as a stress response gene, with its product, the p53 protein, inducing cell cycle arrest or apoptosis in response to DNA damage, thus maintaining genetic stability within the organism (42). Post-stroke, p53 accumulates in the ischemic brain, triggering neuronal apoptosis through transcription-dependent and -independent pathways (43). In response to ischemic insult, p53 swiftly translocates to the mitochondria, where it interacts with Bcl-2 family proteins, thereby activating the mitochondrial apoptotic pathway, with greater efficacy than its role as a transcription factor (44). Ginsenosides, active ingredients of Panax, can inhibit cellular oxidative stress damage by activating miR-30c-5p and suppressing p53, thus ameliorating myocardial ischemia–reperfusion injury (45). Our Western blot analyses were performed to examine the expression of antiapoptotic Bcl-2 and proapoptotic p53 and Bax proteins in the ipsilateral hemispheres containing the ischemic sites. SCLM pretreatment markedly increased the expression of the antiapoptotic protein Bcl-2 while concurrently reducing the expression of the proapoptotic proteins p53 and Bax following MCAO, which is in line with the findings of the aforementioned *in vitro* studies. These observations suggest that SCLM may influence oxidative stress homeostasis in the SD rats subjected to MCAO, thereby mitigating apoptosis and conferring neuroprotection.

Inflammatory cells have dual roles: within the ischemic core, they clear oxidative debris and protect neurons. Conversely, in the ischemic penumbra, the pronounced inflammatory response can exacerbate the infarct area, inhibiting neural repairment and regeneration and ultimately leading to further damage (11, 46). In the ischemic penumbra, microglia function as amplifiers of neuronal injury. The activation of microglia significantly increases both the infarct size and the number of apoptotic neurons, thereby contributing critically to stroke progression (19, 47). TCM has been used to treat inflammatory and related diseases since ancient times. According to the review of abundant modern scientific researches, it is suggested that TCM exhibit anti-inflammatory effects at different levels, and via multiple pathways with various targets (48). For instance, many studies have demonstrated the powerful neuroprotective abilities of multiple traditional Chinese medicines against NLRP3 inflammasome-mediated ischemic cerebral injury (49). Further studies have proved that TCM plays a key role by inhibiting microglial activation, which may be a potential treatment strategy for CNS disorders (50). These TCMs may be in the form of TCM prescriptions, Chinese herbal medicines and their extracts, and TCM monomers. Our findings indicate that the combination of DM+MCAO induces microglial activation, whereas treatment with SCLM can partially attenuate this activation. The regulation of microglial activation is predominantly influenced by several key factors, including the NF-κB signaling pathway, activator protein 1, hypoxia-inducible factor, signal transduction and activation of transcription factors, and peroxisome proliferator-activated receptor gamma, among others (33). Berberine changes the global acetylation landscape in LPS-induced protein acetylation in macrophages and reduces the acetylation of the NF-κB subunit p65 at Lys310 (p65Lys310), leading to the inhibition of NF-κB translocation and transcriptional activity to suppress the expression of inflammatory factors. In this study, we demonstrated that SCLM directly suppresses the expression of key molecules within the TLR4/NF-κB p65 signaling pathway. Furthermore, there was a concomitant reduction in the cytokine IL-1β. These findings underscore the critical role of inflammation regulation in the treatment of diabetes, suggesting that modulating the TLR4/NF-κB signaling pathway constitutes an effective therapeutic strategy for DM+MCAO.

In this study, a DM model with ischemic stroke was established in male SD rats using MCAO combined with STZ. The efficacy of SCLM treatment was subsequently demonstrated. SCLM appears to ameliorate DM with ischemic stroke by modulating oxidative stress and apoptosis in the penumbra region. Additionally, SCLM was found to regulate the NF-κB signaling pathway in microglia. Furthermore, *in vitro* models of PC12 cell hypoxia and BV2 microglial inflammatory activation were established to support these findings. The results of cellular, gene expression and molecular experiments demonstrated that SCLM mitigates oxidative stress and apoptosis through the regulation of the p53/bcl-2/bax signaling pathway. Additionally, SCLM was found to suppress the inflammatory response by modulating the NF-κB signaling pathway. Consequently, this study provides a foundation for the development of therapeutic agents targeting diabetic complications and elucidates the underlying mechanisms of SCLM in the treatment of DM. This study also has certain limitations. First, future studies need to increase the sample size and establish appropriate DM groups and IS groups to distinguish specific drug-disease effects. Second, the relationships between dose and effect are not always linear. These issues will be addressed and elucidated in subsequent experiments.

## 5 Conclusion

**Figure 7:**
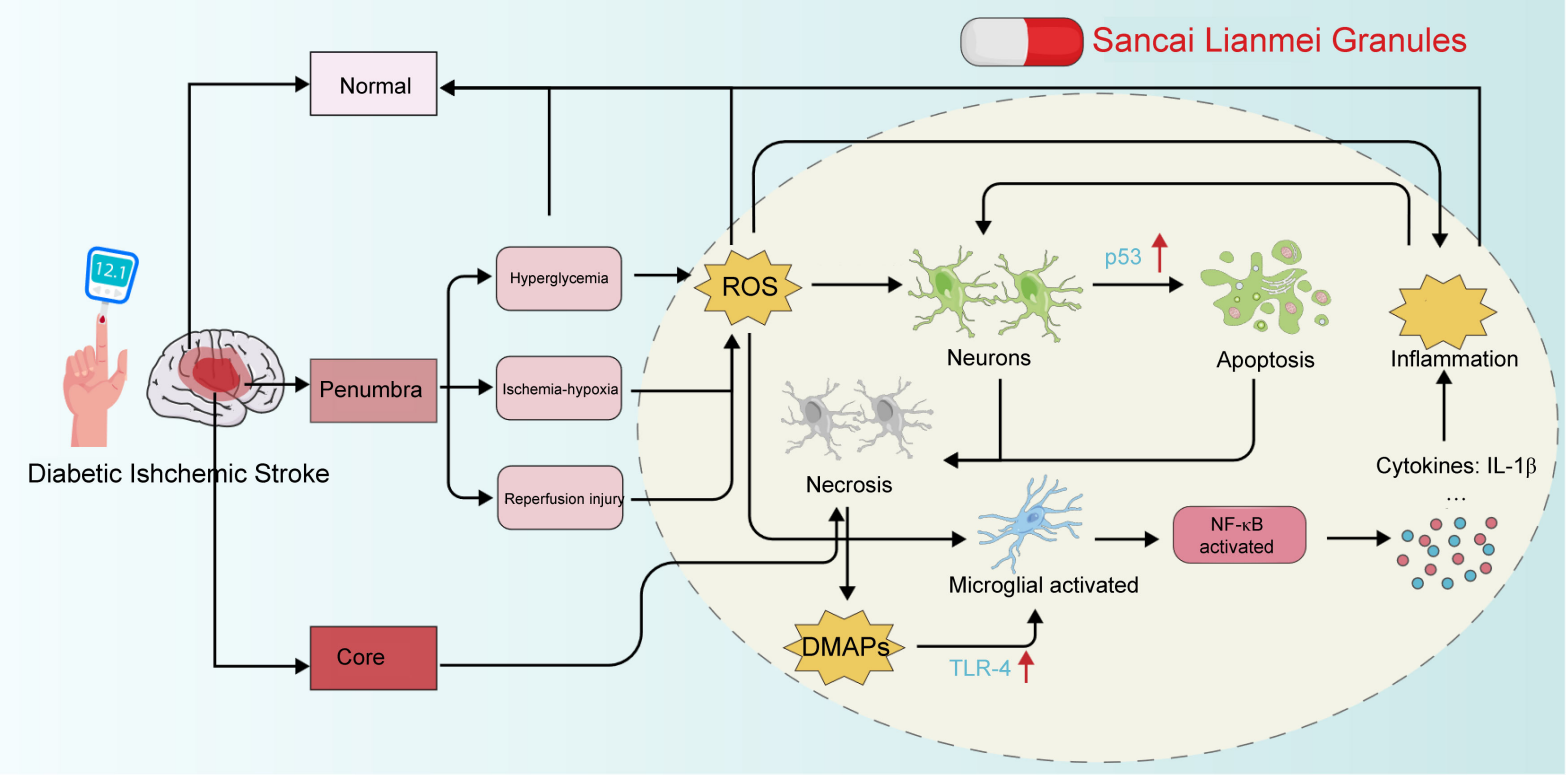
Neuroprotective application mechanisms of SCLM against reperfusion-induced injury in diabetic ischemic stroke.

In this study, we elucidated the anti-ROS and anti-inflammatory mechanisms of SCLM in the context of ischemic stroke concomitant with DM. We identified the underlying pathways through which SCLM upregulates p53, thereby attenuating apoptosis and oxidative stress responses in the penumbra region. Conversely, our findings demonstrate that SCLM inhibits microglial activation by suppressing NF-κB signaling, which subsequently reduces the levels of inflammatory factors (**Figure 7**). Compared to single-mechanism agents like Edaravone (antioxidant) or failed candidates such as Nerinetide, SCLM’s comprehensive approach better addresses stroke’s complex pathophysiology. While preclinical results show advantages in reducing infarct size and improving neurological function, particularly in diabetic models, clinical translation requires further investigation of its pharmacokinetics, standardization, and efficacy in broader stroke populations. The traditional Chinese medicine formulation may offer better safety profiles, but its potential as an adjunct to reperfusion therapy needs rigorous clinical validation.

In conclusion, this discovery may offer theoretical support and a research foundation for the clinical application of SCLM, as well as for the development of therapeutic agents aimed at treating diabetic complications.

## Data Availability

The original contributions presented in the study are included in the article/**Supplementary Material**. Further inquiries can be directed to the corresponding author.
