# Supplementary material for: Sancai Lianmei granules ameliorate neuron injury in diabetic ischemic stroke rats by inhibiting oxidative stress and inflammation
Source: Front Endocrinol (Lausanne). 2025 Aug 27;16:1666597. doi: 10.3389/fendo.2025.1666597 (PMC12420211; doi:10.3389/fendo.2025.1666597)
Supplement: Supplementary file 1 [file DataSheet1.docx]

**Supplementary material**

**Table**

**Table S1** Comparison of Body Wight (BW)

| BW | Control | DM | SCLML | SCLMM | SCLMH | DMBG |
| --- | --- | --- | --- | --- | --- | --- |
| Initial (g) | 275.7±16.7 | 292.8±14.2 | 294.0±12.8 | 294.16±8.2 | 289.7±12.3 | 297.5±10.7 |
| Final (g) | 544±42.1^b^ | 424.9±60.3^a^ | 473.2±54.6^a^ | 453.3±59.8 | 489.5±38.1^a^ | 420.2±58.6 |

^a^*P*＜0.05 versus the control group ^b^*P*＜0.05 versus the DM (model) group

**Table S2.** Comparison of Water Take (WT)

| WT | Control | DM | SCLML | SCLMM | SCLMH | DMBG |
| --- | --- | --- | --- | --- | --- | --- |
| Initial (mL) | 37.5±0.0 | 41.7±7.22 | 41.7±19.1 | 41.7±7.2 | 41.7±7.2 | 41.7±7.2 |
| Final (mL) | 33.3±7.2^b^ | 241.7±47.3^a^ | 129.2±31.5^ab^ | 116.7±40.2^ab^ | 33.3±7.2^abc^ | 129.2±61.7^a^ |

^a^*P*＜0.05 versus the control group; ^b^*P*＜0.05 versus the DM (model) group;

^c^*P*＜0.05 versus the DMBG (positive) group

**Table S3.** Comparison of Blood Glucose (BG)

| BG | Control | DM | SCLML | SCLMM | SCLMH | DMBG |
| --- | --- | --- | --- | --- | --- | --- |
| Initial (mmol/L) | 5.2±0.8 | 4.6±0.6 | 4.9±0.7 | 5.1±0.8 | 4.8±0.8 | 4.6±0.5 |
| Final (mmol/L) | 7.3±1.0^b^ | 26.5±2.9^a^ | 22.6±6.61^a^ | 23.6±5.2^a^ | 16.6±1.9^a^ | 19.7±5.1^ab^ |

^a^*P*＜0.05 versus the control group； ^b^*P*＜0.05 versus the DM(model) group

**Figure**

**Figure S1.** a) NeuN (brown), and DAPI (blue) co-staining of MCAO/R rats brain sections. b) Corresponding semiquantitative analysis of a) (n = 5 per group ) Asterisks indicate *p* values **P* < 0.05, ***P* < 0.01, *****P* < 0.0001, and ns represents no significant difference.


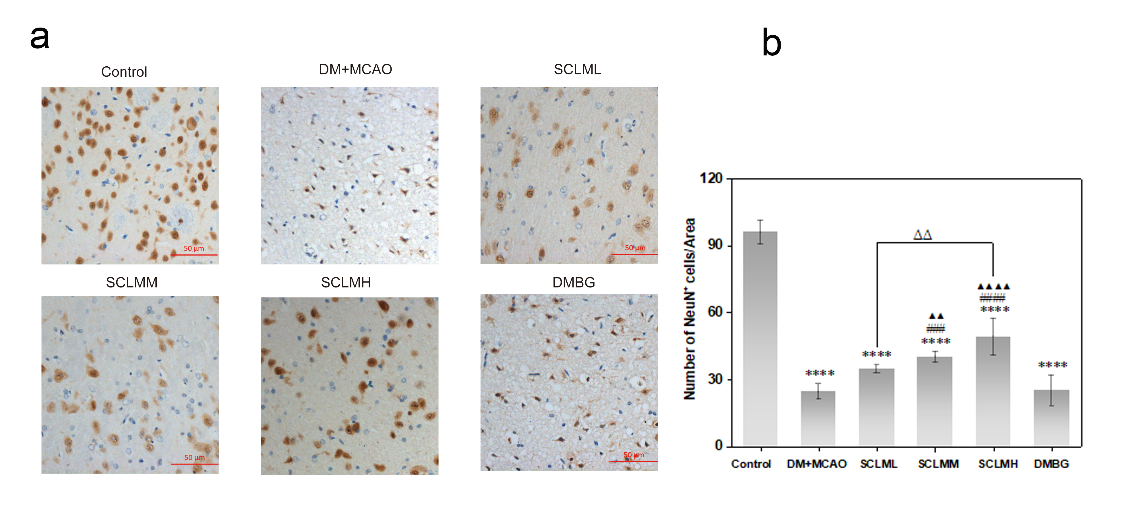


**Figure S2**. a) Cell viability and b) Expression levels of lactate dehydrogenase (LDH) were evaluated following incubation with different concentration of SCLM-contain serum for a duration of 24 hours in PC12. (n=4)


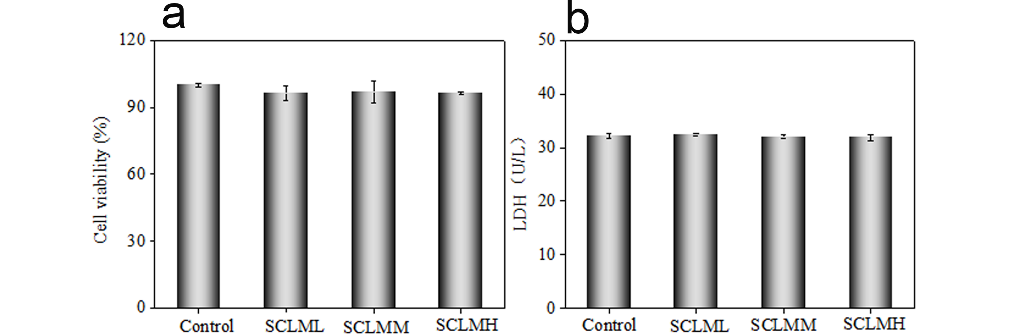


**Figure S3**. Cell viability was evaluated following incubation with different conditions for a duration of 24 hours in PC12. HG25: 25 mM/L high glucose；HG50: 50 mM/L high glucose；HG75: 75 mM/L high glucose；M25: 25 mM/L isotonic mannitol solution; M50: 50 mM/L isotonic mannitol solution; M75: 75 mM/L isotonic mannitol solution. (*n*=6)

**Figure S4**. Cell viability was evaluated following incubation with different oxygen and glucose deprivation (OGD) in PC12. (*n*=10), Asterisks indicate *P* values **P* < 0.05.

**Figure S5**. a) Cell viability and b) Expression levels of lactate dehydrogenase (LDH) were evaluated following incubation with different concentration of SCLM-contain serum for a duration of 24 hours in BV2 cell lines. (*n*=4)
